# European Working Group on Sarcopenia in Older People 2010 (EWGSOP1) and 2019 (EWGSOP2) criteria or slowness: which is the best predictor of mortality risk in older adults?

**DOI:** 10.1093/ageing/afac164

**Published:** 2022-07-23

**Authors:** Maria Claudia Bernardes Spexoto, Paula Camila Ramírez, Roberta de Oliveira Máximo, Andrew Steptoe, Cesar de Oliveira, Tiago da Silva Alexandre

**Affiliations:** Food, Nutrition and Health Postgraduate Program, Federal University of Grande Dourados, Dourados, Brazil; Gerontology Postgraduate Program, Federal University of Sao Carlos, Sao Carlos, Brazil; Escuela de Fisioterapia, Universidad Industrial de Santander, Bucaramanga, Colombia; Physical Therapy Postgraduate Program, Federal University of Sao Carlos, Sao Carlos, Brazil; Physical Therapy Postgraduate Program, Federal University of Sao Carlos, Sao Carlos, Brazil; Department of Epidemiology and Public Health, University College London, London, UK; Department of Epidemiology and Public Health, University College London, London, UK; Gerontology Postgraduate Program, Federal University of Sao Carlos, Sao Carlos, Brazil; Physical Therapy Postgraduate Program, Federal University of Sao Carlos, Sao Carlos, Brazil; Department of Epidemiology and Public Health, University College London, London, UK; Department of Gerontology, Federal University of Sao Carlos, Sao Carlos, Brazil

**Keywords:** sarcopenia, handgrip strength, longitudinal study, English Longitudinal Study of Ageing (ELSA study), mobility, older people

## Abstract

**Objectives:**

to analyse the accuracy of grip strength and gait speed in identifying mortality; to compare the association between mortality and sarcopenia defined by the *EWGSOP1* and *EWGSOP2* using the best cut-off found in the present study and those recommended in the literature and to test whether slowness is better than these two definitions to identify the risk of death in older adults.

**Methods:**

a longitudinal study was conducted involving 6,182 individuals aged 60 or older who participated in the English Longitudinal Study of Ageing. Sarcopenia was defined based on the *EWGSOP1* and *EWGSOP2* using different cut-off for low muscle strength (LMS). Mortality was analysed in a 14-year follow-up.

**Results:**

compared with the LMS definitions in the literature (<32, <30, <27 and < 26 kg for men; <21, <20 and < 16 kg for women), the cut-off of <36 kg for men (sensitivity = 58.59%, specificity = 72.96%, area under the curve [AUC] = 0.66) and < 23 kg for women (sensitivity = 68.90%, specificity = 59.03%, AUC = 0.64) as well as a low gait speed (LGS) ≤0.8 m/s (sensitivity = 53.72%, specificity = 74.02%, AUC = 0.64) demonstrated the best accuracy for mortality. Using the cut-off found in the present study, probable sarcopenia [HR = 1.30 (95%CI: 1.16–1.46)], sarcopenia [HR = 1.48 (95%CI: 1.24–1.78)] and severe sarcopenia [HR = 1.78 (95%CI: 1.49–2.12)] according to *EWGSOP2* were better predictors of mortality risk than *EWGSOP1.* LGS ≤0.8 m/s was a better mortality risk predictor only when LMS was defined by low cut-off.

**Conclusions:**

using LMS <36 kg for men and < 23 kg for women and LGS ≤ 0.8 m/s, *EWGSOP2* was the best predictor for mortality risk in older adults.

## Key Points

- Low muscle strength (LMS) defined as a handgrip strength (HGS) <36 kg for men and < 23 kg for women and low gait speed (LGS) defined as gait speed (GS) ≤0.8 m/s showed the best accuracy for mortality.
- When low muscle strength (LMS) is defined as handgrip strength (HGS) <36 kg for men and < 23 kg for women and low gait speed (LGS) is defined as gait speed (GS) ≤0.8 m/s, European Working Group on Sarcopenia in Older People (*EWGSOP2*) is a better predictor for mortality risk in older adults.
- Low gait speed (LGS) ≤0.8 m/s is a better mortality risk predictor only when low muscle strength (LMS) is defined using low cut-off.

## Introduction

Two operational definitions for sarcopenia were proposed by the European Working Group on Sarcopenia in Older People (EWGSOP): 2010 (*EWGSOP1*) [1] and 2019 (*EWGSOP2*) [2]. The *EWGSOP1* proposed the diagnosis of sarcopenia in the presence of low muscle mass (LMM) and low muscle function determined by the reduction in muscle strength or physical performance. Severe sarcopenia was defined when the three components were present [1]. The *EWGSOP2* proposed the diagnosis of sarcopenia by the combination of low muscle strength (LMS) and LMM. Physical performance, which was previously a central component of the definition, categorised the severity of the condition in the new definition. However, the two guidelines have little diagnostic agreement, generating discrepancies in the prevalence of sarcopenia, which ranges from 11 to 27.7% when the *EWGSOP1* is applied and 4.6 to 13.6% when the *EWGSOP2* is applied [3–7].

Recent studies comparing these definitions have found an association with a greater risk of mortality when sarcopenia is defined by the *EWGSOP1* but not when defined by the *EWGSOP2* [4, 8–10]. One explanation for this disagreement may be the inclusion of gait speed (GS) in the assessment, which was previously part of the diagnosis of sarcopenia and not only an indicator of its severity. Another explanation for this divergence may reside in the use of lower cut-off points for LMS, which is a primary parameter in the *EWGSOP2.* This argument is supported by the use of different cut-off for handgrip strength (HGS) reported in the literature for different outcomes [11–14].

Lauretani *et al*. [12] demonstrated that a HGS <30 kg for men and < 20 kg for women was associated with mobility limitation. However, the authors did not report sensitivity, specificity or other components of accuracy analysis. Alley *et al*. [14] found that HGS <26 kg for men (23.4% sensitivity and 96.6% specificity) and < 16 kg for women (30.6% sensitivity and 87.5% specificity) were the best indicators of weakness associated with mobility limitation. More recently, Delinocente *et al*. [13] found that a HGS <32 kg for men (49.1% sensitivity, 79.8% specificity and area under the curve of 0.82) and < 21 kg for women (58.6% sensitivity, 72.9% specificity and area under the curve of 0.83) were the best cut-off for identifying mobility limitation. Furthermore, the *EWGSOP2* recommends HGS <27 kg for men and < 16 kg for women for LMS; these cut-off were obtained based on population distribution rather than diagnostic accuracy analysis [2], and were tested by Costanzo *et al*. to identify a 3-year follow-up mortality risk with 48% of sensitivity and 84% of specificity [15].

However, the accuracy of HGS and GS to define mortality risk in a sarcopenia context using a long follow-up period has not been tested. Furthermore, no study has compared the two definitions testing different cut-off for LMS or analysed the importance of LGS to the diagnosis of sarcopenia and its association with mortality. Therefore, the aims of the present study were (i) To analyse the accuracy of HGS and GS to identify mortality risk; (ii) To compare the association between mortality and sarcopenia defined by the *EWGSOP1* and *EWGSOP2* using the cut-off found in the present study and those recommended in the literature (<32, <30, <27 and < 26 kg for men; <21, <20 and < 16 kg for women) and (iii) To verify whether slowness is better than these two definitions to identify the risk of death in older adults.

## Methods

### Study population

Data were extracted from the English Longitudinal Study of Ageing (ELSA), which is a panel study started in 2002 with a representative sample of community-dwelling English men and women aged 50 years or older [16]. Details on the ELSA methods can be found in a previous publication [17].

We used the second wave of the ELSA study (2004) as the baseline, which is when anthropometric measures and physical performance were investigated for the first time, involving 6,182 participants aged 60 years or older.

### Muscle strength assessment

HGS was measured using a dynamometer. HGS was analysed as a continuous variable in the accuracy analysis. In the mortality analyses, LMS was considered when HGS was <32, <30, <27 and < 26 kg for men and < 21, <20 and < 16 kg for women [1, 2, 11–14]. Detailed information can be found in the Supplemental Material (Section Muscle strength assessment).

### Appendicular skeletal muscle mass assessment

Appendicular skeletal muscle mass (ASMM) was determined using the Lee equation [18, 19]. In a study investigating the association between multimorbidity at baseline and the onset of sarcopenia over 12 years of follow-up in a large representative sample of the English older adult population, Veronese *et al*. also used this equation to estimate ASMM [20]. LMM was considered when the ASMMI was <9.24 kg/m^2^ for men and < 6.52 kg/m^2^ for women [21, 22]. Detailed information can be found in the Supplemental Material (Section Appendicular skeletal muscle mass assessment).

### Physical performance

GS was used for the assessment of physical performance [23, 24]. GS was analysed as a continuous variable in the accuracy analysis. In the mortality analyses, GS ≤0.8 m/s was considered to have LGS [1, 2]. Detailed information can be found in the Supplemental Material (Section Physical performance).

All measures used for the definition and diagnosis of sarcopenia were taken at baseline.

### Definition and diagnosis of sarcopenia

The criteria proposed by the *EWGSOP1* [1] and *EWGSOP2* [2] were used for the definition of sarcopenia. Detailed information can be found in the Supplemental Material (Section Definition and diagnosis of sarcopenia).

### Covariates

The covariates included in the present analysis constitute a broad spectrum of factors associated with mortality [8, 10, 25] as sex, age, total household wealth, marital status, level of education [26, 27], smoking status, alcohol intake, physical activity level [26, 28, 29], self-report of systemic arterial hypertension, diabetes, cancer, lung disease, heart disease, stroke, falls, depressive symptoms [30], memory status [31], number of medications and abdominal obesity [32, 33]. Detailed information can be found in the Supplemental Material (Section Covariates).

### Mortality

Mortality data were obtained from the Office for National Statistics of England.

### Statistical analysis

We imputed missing data due to item non-response using multiple imputation by chained equations, which included all variables (including the survival outcome) in the prediction model to generate 20 imputed datasets (each had a final *n* = 6,182) [34]. Owing to the greater precision offered, we present the analyses from the imputed datasets in this paper. We have also used longitudinal weights in all models.

Sensitivity, specificity, log-likelihood positive (LR+), negative (LR−), area under the receiver operating characteristic curves and Youden Index values were calculated to determine the accuracy of HGS and GS in identifying mortality [35, 36].

The sample characteristics at baseline were expressed as mean, standard deviation and proportion. We examined all deaths occurred in the 14-year follow-up period. The follow-up time was defined by the date of the last visit/interview and the date of death. The time for those who deceased was calculated by the difference between the date of death (day/month/year) and date of the oldest interview. The time for those who lived through the end of the follow-up period was calculated by the difference between the last data recorded (visit/interview) and the data from the baseline interview.

Survival curves were analysed using the Kaplan–Meier method to explore the association between the different definitions of sarcopenia and mortality. Differences between curves were evaluated using the log-rank test.

Based on proportional risk models, Cox regression analysis was applied to explore the association between sarcopenia and mortality. For such, unadjusted and adjusted hazard ratios (HRs) and respective 95% confidence intervals (CIs) were estimated. The adjusted models were controlled for all sociodemographic, behavioural, clinical and anthropometric variables.

The definitions of sarcopenia according to the *EWGSOP1* and *EWGSOP2* were constructed with different cut-off points for HGS. The diagnosis proposed by the ***EWGSOP1*** was used for Constructs 1–4, as follows: *Construct 1:* LMS <26/16 kg; *Construct 2:* LMS <27/16 kg; *Construct 3:* LMS <30/20 kg; *Construct 4:* LMS <32/21 kg. The diagnosis proposed by the ***EWGSOP2*** was used for Constructs 6–9, as follows: *Construct 6:* LMS <26/16 kg; *Construct 7:* LMS <27/16 kg; *Construct 8:* LMS <30/20 kg; *Construct 9:* LMS <32/21 kg.

LMM, LMS and LGS as isolated conditions were also analysed to identify which had a stronger association with an increased risk of mortality.

All models were compared using the concordance index or C-index. A C-index of 0.5 indicates a poor performing model, whereas a value of 1 indicates a model with perfect prediction [37, 38].

The *Stata* 16.1® statistical package was used for all analyses, with a *P*-value <0.05 considered indicative of statistical significance.

## Results

**Figure 1:**
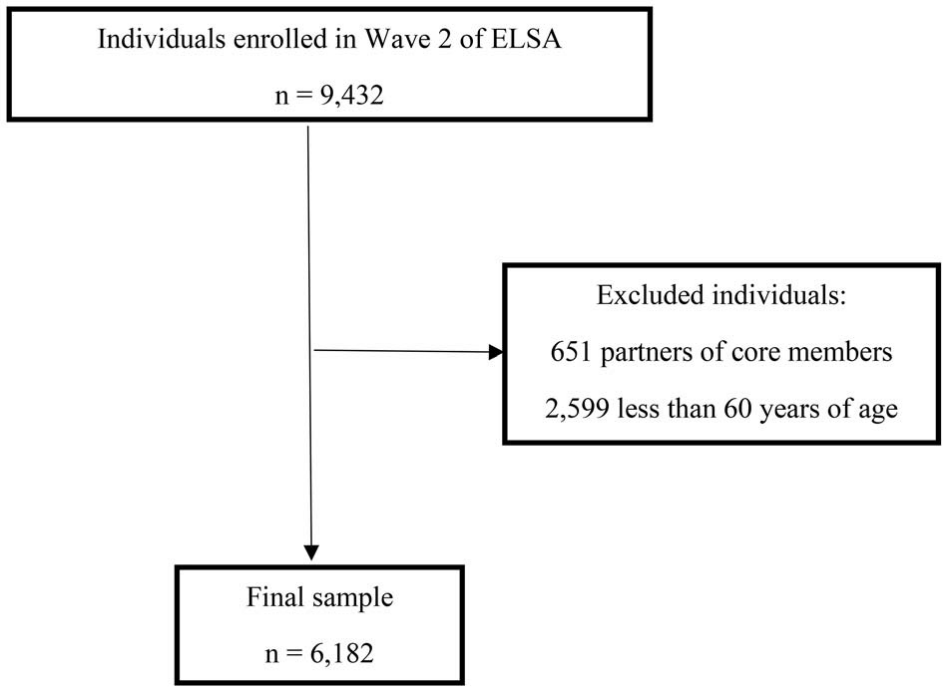
Flowchart of participant selection process.

Among the 6,182 participants of the study (Figure 1), 2,669 died in the 14-year follow-up period. The sociodemographic, behavioural, clinical and anthropometric characteristics and components of sarcopenia of the participants at baseline are displayed in Table 1.

**Table 1:** Baseline characteristics of 6,182 older adults participating in ELSA study (2004)

**Variables**	**ELSA *n* = 6,182**
**Sociodemographic characteristics**	
Age, years	71.4 ± 7.9
Age groups, (%)	
60–69	46.5
70–79	35.4
≥ 80	18.1
Sex (female), (%)	55.5
Marital status (with conjugal life), (%)	63.1
Total household wealth, (%)	
1st quintile (highest quintile)	20.0
2nd quintile	19.6
3rd quintile	19.9
4th quintile	20.0
5th quintile (lowest quintile)	19.5
Not reported, (%)	1.0
Educational level, (%)	
> 13 years	20.6
12–13 years	19.3
0–11 years	60.1
**Behavioural characteristics**	
Smoking status, (%)	
Never smoked	36.0
Former smoker	51.2
Current smoker	12.8
Alcohol intake, (%)	
Non-drinker or rare drinker	19.5
Frequent drinker	37.1
Daily drinker	28.5
Did not answer	14.9
Sedentary lifestyle, (%)	42.3
**Clinical characteristics**	
Arterial hypertension (yes), (%)	48.7
Diabetes (yes), (%)	10.2
Cancer (yes), (%)	9.2
Lung disease (yes), (%)	18.8
Heart disease (yes), (%)	26.9
Stroke (yes), (%)	6.9
Falls (yes), (%)	32.9
Depressive symptoms (yes), (%)	15.5
Memory Score, points	9.1 ± 3.7
Medications, number	0.5 ± 0.7
**Waist circumference, (%)**	
Non-abdominal obesity	47.6
Abdominal obesity	52.4
Waist circumference, cm	
Men	101.7 ± 12.1
Women	91.0 ± 13.5
**Components of sarcopenia**	
Grip strength, kg	
Men	36.9 ± 10.4
Women	21.9 ± 7.1
LMS (< 26 kg ♂; < 16 kg ♀), (%)	15.2
LMS (< 27 kg ♂; < 16 kg ♀), (%)	16.3
LMS (< 30 kg ♂; < 20 kg ♀), (%)	28.4
LMS (< 32 kg ♂; < 21 kg ♀), (%)	36.2
LMS (< 36 kg ♂; < 23 kg ♀), (%)	49.6
Appendicular skeletal muscle mass index, kg/m^2^	
Men	10.1 ± 1.2
Women	7.6 ± 1.5
LMM, (%)	19.9
GS, m/s	
Men	0.91 ± 0.3
Women	0.84 ± 0.3
Low gait speed (≤ 0.8 m/s), (%)	40.9

Data expressed as proportion, mean and standard deviation.

The sample was composed predominantly of women (55.5%) as well as individuals with a conjugal life (63.1%) and low schooling (0–11 years) (60.1%). Regarding behavioural characteristics, most were former-smokers (51.2%) with an active lifestyle (57.7%) and 37.1% reported frequent alcohol intake. Among the chronic diseases investigated, systemic arterial hypertension was the most prevalent (48.7%), followed by heart disease (26.9%). The majority had abdominal obesity (52.4%) (Table 1).

LMS defined as HGS <36 kg for men (sensitivity = 58.59%, specificity = 72.96%, LR+ = 2.17, LR− = 0.57, AUC = 0.66 and Youden = 0.32) and < 23 kg for women (sensitivity = 68.90%, specificity = 59.03%, LR+ = 1.68, LR− = 0.53, AUC = 0.64 and Youden = 0.28) was more accurate in identifying mortality (Table 2). LGS defined as GS ≤0.8 m/s (sensitivity = 53.72%, specificity = 74.02%, LR+ = 2.07, LR− = 0.62, AUC = 0.64 and Youden = 0.28) was more accurate in identifying mortality, confirming what is recommended by the consensus (Table 3). Based on these cut-off points, *Construct 5* (*EWGSOP1* with LMS <36/23 kg) and *Construct 10* (*EWGSOP2* com LMS <36/23 kg) were created.

**Table 2:** Diagnostic properties of HGS cut-off to identify mortality (ELSA study)

**Cut-off (kg)**	**Sensitivity**	**Specificity**	**Correct Classification**	**LR+**	**LR−**	**AUC**	**Youden**
**Men**							
<24	11.17	95.96	58.74	2.76	0.93	0.54	0.07
<25	13.28	95.05	59.16	2.68	0.91	0.54	0.08
<26	15.49	94.31	59.71	2.72	0.90	0.55	0.10
<27	18.55	92.91	60.27	2.62	0.88	0.56	0.11
<28	20.86	92.17	60.87	2.66	0.86	0.56	0.13
<29	24.45	90.85	61.70	2.67	0.83	0.58	0.15
<30	26.98	89.53	62.07	2.58	0.82	0.58	0.17
<31	34.25	87.14	63.92	2.66	0.75	0.61	0.21
<32	38.78	84.91	64.66	2.57	0.72	0.62	0.24
<33	43.73	82.36	65.40	2.48	0.68	0.63	0.26
<34	47.63	79.72	65.63	2.35	0.66	0.64	0.27
<35	52.37	77.08	66.23	2.28	0.62	0.65	0.29
<36	58.59	72.96	66.65	2.17	0.57	0.66	0.32
<37	62.38	67.68	65.36	1.93	0.56	0.65	0.30
<38	67.02	63.64	65.12	1.84	0.52	0.65	0.31
<39	70.71	59.52	64.43	1.75	0.49	0.65	0.30
**Women**							
<11	5.62	97.02	64.92	1.88	0.97	0.51	0.03
<12	7.56	96.20	65.07	1.99	0.96	0.52	0.04
<13	10.80	95.32	65.64	2.31	0.94	0.53	0.06
<14	13.50	94.27	65.91	2.36	0.92	0.54	0.08
<15	17.49	92.93	66.44	2.47	0.89	0.55	0.10
<16	23.65	90.77	67.20	2.56	0.84	0.57	0.14
<17	28.73	87.55	66.89	2.31	0.81	0.58	0.16
<18	34.67	84.98	67.31	2.31	0.77	0.60	0.20
<19	42.76	81.30	67.77	2.29	0.70	0.62	0.24
<20	47.08	78.14	67.24	2.15	0.68	0.63	0.25
<21	55.94	70.95	65.68	1.93	0.62	0.63	0.27
<22	61.45	65.05	63.78	1.76	0.59	0.63	0.27
<23	68.90	59.03	62.50	1.68	0.53	0.64	0.28
<24	73.97	53.24	60.52	1.58	0.49	0.64	0.27
<25	79.27	46.52	58.02	1.48	0.45	0.63	0.26
<26	85.53	38.81	55.21	1.40	0.37	0.62	0.24
<27	88.77	31.74	51.76	1.30	0.35	0.60	0.21
<28	91.14	26.07	48.92	1.23	0.34	0.59	0.17
<29	93.20	20.86	46.26	1.18	0.33	0.57	0.14
<30	84.82	16.13	43.76	1.13	0.32	0.55	0.01

LR+: positive Log-likelihood; LR−: negative Log-likelihood; AUC: area under curve

Regarding the components of sarcopenia, 49.6%, 36.2%, 28.4%, 16.3% and 15.2% had LMS using the cut-off of <36, <32, <30, <27 and < 26 kg for men and < 23, <21, <20 and < 16 kg for women, respectively. LMM was found in 19.9% and LGS was found in 40.9% of the individuals (Table 1).

Higher prevalence values for sarcopenia were found when the LMS cut-off were < 36/23 kg for both *EWGSOP1* and *EWGSOP2*. However, *EWGSOP2* was better in identifying sarcopenia with 33.9% probable sarcopenia, 6.2% sarcopenia and 8.6% severe sarcopenia (Table 4).

Associations between sarcopenia status and mortality using the *EWGSOP1* and *EWGSOP2* definitions are presented in Table 4 and Supplemental Table 1. *EWGSOP2* using the cut-off of <36/23 kg to define LMS was the best mortality risk predictor in older adults in 14-year follow-up period. In the completely adjusted models, individuals with probable sarcopenia showed 30% higher mortality risk than non-sarcopenic. This was the only significant cut-off value for probable sarcopenia. The mortality risk increased to 48% for those with sarcopenia and 78% for those with severe sarcopenia (Table 4).

Table 5 shows the analysis of the sarcopenia components separately. A LGS ≤ 0.8 m/s was a better mortality risk predictor only when LMS was defined using low cut-off values. When the cut-off <36/23 kg were used, the mortality risk for those individuals with LMS was 35% (95%CI 1.22–1.49) higher than those with HGS ≥36/23 kg and 36% (95%CI 1.23–1.50) higher for those with LGS than those with GS >0.8 m/s i.e. the mortality risks were practically the same.

**Table 3:** Diagnostic properties of GS cut-off to identify mortality (ELSA study)

**Cut-off (m/s)**	**Sensitivity**	**Specificity**	**Correct Classification**	**LR+**	**LR**−	**AUC**	**Youden**
≤0.3	3.80	99.38	62.09	6.16	0.97	0.52	0.03
≤0.4	8.51	98.39	63.32	5.29	0.93	0.53	0.07
≤0.5	16.32	96.34	65.11	4.45	0.87	0.56	0.13
≤0.6	27.18	91.82	66.60	3.32	0.79	0.59	0.19
≤0.7	40.29	84.77	67.41	2.64	0.70	0.62	0.25
≤0.8	53.72	74.02	66.10	2.07	0.62	0.64	0.28
≤0.9	68.43	59.74	63.13	1.70	0.53	0.64	0.28
≤1.0	80.10	44.03	58.10	1.43	0.45	0.62	0.24
≤1.1	88.76	30.64	53.32	1.28	0.37	0.60	0.19
≤1.2	93.69	18.76	48.00	1.15	0.34	0.56	0.12
≤1.3	96.74	10.75	44.30	1.08	0.30	0.54	0.07
≤1.4	98.13	6.03	41.96	1.04	0.31	0.52	0.04
≤1.5	98.88	3.25	40.56	1.02	0.34	0.51	0.02
≤1.6	99.41	1.88	39.94	1.01	0.31	0.51	0.01

lr+: positive log-likelihood; lr−: negative log-likelihood; auc: area under curve

## Discussion

We found that LMS <36 kg for men and < 23 kg for women and LGS ≤0.8 m/s were more accurate in identifying mortality. *EWGSOP2* using these cut-off was more accurate in identifying mortality risk than *EWGSOP1.* In addition, LGS ≤0.8 m/s was a better predictor of mortality risk only when lower LMS cut-off were used.

Petermann-Rocha *et al*. [8], Locquet *et al*. [4, 39], Sobestiansky *et al*. [9], Costanzo *et al*. [15], Phu *et al*. [7], Reiss *et al*. [3], and Yang *et al*. [5], cross-sectionally and longitudinally, and a recent systematic review by Fernandes *et al*. [40] found that the prevalence of sarcopenia by *EWGSOP2* was considerably lower than *EWGSOP1*. In all the studies aforementioned, *EWGSOP2* was used with lower LMS cut-off and, as a result, a lower prevalence of sarcopenia was reported. Our findings corroborate previous findings in relation to the use of low HGS cut-off to define LMS. However, the prevalence of sarcopenia is higher and more similar between *EWGSOP1* and *EWGSOP2* when both define LMS as HGS <36/23 kg.

With regards to the association between sarcopenia, defined by *EWGSOP2*, and higher mortality risk, previous studies showed conflicting results which could be attributed to how probable sarcopenia, sarcopenia and severe sarcopenia were analysed, length of follow-up, age and type of participants. For example, Petermann-Rocha *et al*. [8], analysing 469,858 *UK Biobank* community-dwelling participants aged between 40 and 69 and followed-up for 2 years, found that sarcopenia [(LMS <27 kg for men and < 16 kg for women) + (LMM <7.0 kg/m^2^ for men and < 5.5 kg/m^2^ for women)] was not associated with mortality risk (HR = 1.25 CI 95% 0.99–1.58). Sobestiansky *et al*. [9], analysing data from 287 community-dwelling men aged between 85 and 89 of the *Uppsala Longitudinal Study of Adult Men* (ULSAM) during a 3-year follow-up, also did not find an association between sarcopenia defined as LMS <27 kg + LMM <7.0 kg/m^2^ or as LMS <26 Kg + LMM <7.0 kg/m^2^ and mortality (HR = 1.70 95%CI 0.94–3.05 and HR = 1.65 95%CI 0.94–3.05), respectively. Costanzo *et al*. [15], analysing 535 participants of the *InCHIANTI* study aged 65 or older followed-up for 3 years, did not find an association between sarcopenia [(LMS <27 kg for men and < 16 kg for women) + (LMM <7.0 kg/m^2^ for men and < 6.0 kg/m^2^ for women)] and mortality (HR = 1.96 95%CI 0.63–6.15). Finally, Bachettini *et al*. [10], analysing 1,291 community-dwelling individuals aged 60 or older and followed-up for 2.6 years, also did not find an association between sarcopenia [(LMS <29.7 kg for men and < 16.2 kg for women) + (LMM ≤34 cm of calf circumference for men and ≤ 33 cm for women)] and mortality (HR = 1.36 95%CI 0.52–3.57).

**Table 4:** Prevalence and cox proportional hazard for different constructs of sarcopenia to predict mortality in 14-year follow-up among 6,182 older adults from ELSA study

**Prevalence (%) (95% CI)**										
	**Construct 1**	**Construct 2**	**Construct 3**	**Construct 4**	**Construct 5**	**Construct 6**	**Construct 7**	**Construct 8**	**Construct 9**	**Construct 10**
No sarcopenia	78.8 (77.5–79.9)	78.8 (77.5–79.9)	78.8 (77.5–79.9)	78.8 (77.5–79.9)	78.8 (77.5–79.9)	86.3 (85.3–87.3)	85.2 (84.3–86.2)	73.0 (71.7–74.2)	65.0 (63.7–66.3)	51.3 (49.8–52.7)
Pre-sarcopenia^1^	9.9 (9.0–10.7)	9.8 (8.9–10.6)	8.4 (7.6–9.2)	7.2 (6.4–8.0)	5.1 (4.5–5.8)	9.0 (8.1–9.9)	9.6 (8.7–10.4)	18.2 (17.1–19.3)	23.6 (22.3–24.9)	33.9 (32.6–35.2)
Sarcopenia^2^	7.6 (6.8–8.4)	7.4 (6.6–8.2)	6.7 (6.0–7.6)	6.8 (6.1–7.7)	7.7 (6.9–8.5)	1.2 (0.9–1.6)	1.4 (1.1–1.7)	2.8 (2.3–3.3)	4.2 (3.6–4.7)	6.2 (5.5–6.9)
Severe Sarcopenia	3.7 (3.2–4.2)	4.0 (3.5–4.5)	6.1 (5.4–6.7)	7.2 (6.5–7.9)	8.4 (7.7–9.2)	3.5 (2.9–4.0)	3.8 (3.2–4.4)	6.0 (5.2–6.7)	7.2 (6.5–8.0)	8.6 (7.8–9.3)
**Adjusted Model HR (95% CI)**										
	**Construct 1**	**Construct 2**	**Construct 3**	**Construct 4**	**Construct 5**	**Construct 6**	**Construct 7**	**Construct 8**	**Construct 9**	**Construct 10**
No sarcopenia	1.00	1.00	1.00	1.00	1.00	1.00	1.00	1.00	1.00	1.00
Pre-sarcopenia^1^	1.11 (0.94–1.32)	1.10 (0.93–1.31)	1.11 (0.91–1.34)	1.07 (0.86–1.33)	0.95 (0.71–1.28)	1.06 (0.90–1.24)	1.03 (0.90–1.18)	1.08 (0.95–1.23)	1.12 (1.00–1.26)	1.30 (1.16–1.46)
Sarcopenia^2^	1.37 (1.17–1.61)	1.38 (1.17–1.63)	1.32 (1.12–1.56)	1.26 (1.06–1.49)	1.23 (1.05–1.45)	1.02 (0.73–1.42)	1.06 (0.77–1.45)	1.08 (0.86–1.36)	1.21 (0.99–1.48)	1.48 (1.24–1.78)
Severe Sarcopenia	1.36 (1.11–1.67)	1.36 (1.12–1.65)	1.37 (1.16–1.61)	1.43 (1.22–1.68)	1.48 (1.27–1.73)	1.21 (0.97–1.50)	1.19 (0.95–1.50)	1.29 (1.08–1.53)	1.44 (1.21–1.71)	1.78 (1.49–2.12)
*C-index*	0.7779	0.7780	0.7777	0.7781	0.7785	0.7765	0.7765	0.7771	0.7779	0.7794

HR: hazard ratio; CI: confidence interval. Models adjusted for sex, age, total household wealth, marital status, smoking status, alcohol intake, physical activity level, systemic arterial hypertension, diabetes, cancer, lung disease, heart disease, stroke, falls in the previous year, depressive symptoms, memory performance, number of medications and waist circumference; ^1^ probable sarcopenia in *EWGSOP2*; ^2^ or confirmed sarcopenia in *EWGSOP2*

***EWGSOP1*:**

Construct 1—LMS <26/16 kg; Construct 2—LMS <27/16 kg; Construct 3—LMS <30/20 kg; Construct 4—LMS <32/21 kg; Construct 5—LMS <36/23 kg

***EWGSOP2*:**

Construct 6—LMS <26/16 kg; Construct 7—LMS <27/16 kg; Construct 8—LMS <30/20 kg; Construct 9—LMS <32/21 kg; Construct 10—LMS <36/23 kg

On the other hand, Malafarina *et al*. [41], analysing 187 individuals with an average age of 85 years undergoing post-surgical hip fracture rehabilitation during a 7 follow-up period, found an association between sarcopenia [(LMS <27 kg for men and < 16 kg for women) + (LMM <7.0 kg/m^2^ for men and < 6.0 kg/m^2^ for woman)] and mortality (HR = 1.67 95%CI 1.11–2.51). Bianchi *et al*. [42], analysing 527 hospitalised individuals with an average age of 80 years after a 3-year follow-up, also found an association between sarcopenia [(LMS <27 kg for men and < 16 kg for women) + (LMM <7.0 kg/m^2^ for men and < 5.5 kg/m^2^ for women)] and mortality (HR = 1.84 95%CI 1.33–2.57).

However, despite the conclusions from a recent meta-analysis involving 42,108 individuals aged 49 and older showing that sarcopenia is associated to a higher mortality risk, independent of the type of population investigated, sarcopenia definition, length of follow-up and risk of bias, only five studies using the *EWGSOP2* definition were included with very conflicting results [25]. Therefore, when our findings are compared with the ones from previous studies, the use of higher LMS cut-off in the *EWGSOP2* definition to identify community-dwelling older adults with sarcopenia is a better approach not only in terms of estimating prevalence but also to identify mortality risk. Such approach could be very useful to promote preventive strategies and treatment. It is likely that the identification of the best cut-off value in our study was because ELSA has a long follow-up period. Lower cut-off in combination with short follow-up periods showed in the literature are more useful in hospital settings.

Our key findings also highlighted that slowness, separately, was the best mortality risk predictor only when lower LMS cut-off were used (<32, 30, 27 and 26 kg for men and < 21, 20 and 16 kg for women). When the cut-off <36/23 kg were tested, the mortality risk for those who had LMS and LGS compared with those with normal HGS and GS was practically the same. However, despite these two components having very similar mortality risks, their order of entry in the flowchart of the two consensuses on sarcopenia modified the results of the association between sarcopenia status and mortality. It is better when the initial identification process is done with muscle strength before GS, which confirms the advantage of using the algorithm proposed by *EWGSOP2*.

This study has limitations and strengths that should be acknowledged. Our findings should be considered in the context of community-dwelling individuals aged 60 years or older. Caution should be exercised regarding the interpretation of the results in the clinical/hospital setting and in nursing homes. Another important limitation regards the determination of ASMM using an equation. However, this does not invalidate our findings, as the equation has been validated using gold-standard methods, such as magnetic resonance and dual-energy X-ray absorptiometry. This study also has strong points, such as the inclusion of a large representative sample of community-dwelling older English adults, a 14-year follow-up period and the fact that it is the first study to compare the association of sarcopenia defined by the *EWGSOP1* and *EWGSOP2* using different cut-off for HGS recommended in the literature and proposed by the present study. Moreover, our survival analysis was adjusted for a wide range of covariates associated with mortality.

**Table 5:** Cox proportional hazard of sarcopenia components predicting mortality in 14-year follow-up among 6,182 older adults from ELSA study

	**Unadjusted model HR (95% CI)**	**Adjusted model HR (95% CI)**
**References: Normal muscle strength/normal muscle mass/normal GS**	1.00	1.00
**Model 1**		
LMS (< 26/16 kg)	1.62 (1.47–1.80)	1.10 (0.99–1.24)
LMM (< 6.52 kg/m^2^; < 9.24 kg/m^2^)	2.01 (1.83–2.21)	1.24 (1.09–1.42)
Low gait speed (≤ 0.8 m/s)	2.54 (2.33–2.76)	1.39 (1.26–1.54)
*C-index*	0.6606	0.7789
**Model 2**		
LMS (< 27/16 kg)	1.63 (1.47–1.80)	1.08 (0.97–1.21)
LMM (< 6.52 kg/m^2^; < 9.24 kg/m^2^)	2.00 (1.82–2.19)	1.24 (1.09–1.42)
Low gait speed (≤ 0.8 m/s)	2.53 (2.32–2.75)	1.39 (1.26–1.54)
*C-index*	0.6618	0.7789
**Model 3**		
LMS (< 30/20 kg)	1.70 (1.55–1.86)	1.14 (1.03–1.26)
LMM (< 6.52 kg/m^2^; < 9.24 kg/m^2^)	1.94 (1.76–2.13)	1.23 (1.08–1.40)
Low gait speed (≤ 0.8 m/s)	2.41 (2.21–2.62)	1.38 (1.25–1.52)
*C-index*	0.6679	0.7790
**Model 4**		
LMS (< 32/21 kg)	1.81 (1.66–1.98)	1.16 (1.04–1.28)
LMM (< 6.52 kg/m^2^; < 9.24 kg/m^2^)	1.90 (1.73–2.09)	1.23 (1.08–1.41)
Low gait speed (≤ 0.8 m/s)	2.35 (2.16–2.56)	1.38 (1.25–1.52)
*C-index*	0.6724	0.7791
**Model 5**		
LMS (< 36/23 kg)	2.09 (1.91–2.29)	1.35 (1.22–1.49)
LMM (< 6.52 kg/m^2^; < 9.24 kg/m^2^)	1.86 (1.69–2.04)	1.23 (1.08–1.39)
Low gait speed (≤ 0.8 m/s)	2.32 (2.14–2.52)	1.36 (1.23–1.50)
*C-index*	0.6829	0.7800

HR: hazard ratio; CI: confidence interval. Models adjusted for sex, age, total household wealth, marital status, smoking status, alcohol intake, physical activity level, systemic arterial hypertension, diabetes, cancer, lung disease, heart disease, stroke, falls in the previous year, depressive symptoms, memory performance, number of medications and waist circumference.

Model 1: HGS <26/16 kg.

Model 2: HGS <27/16 kg.

Model 3: HGS <30/20 kg.

Model 4: HGS <32/21 kg.

Model 5: HGS <36/23 kg.

## Conclusion

LMS <36 kg for men and < 23 kg for women and LGS ≤0.8 m/s demonstrated best accuracy for mortality. LMS <36/23 kg and LGS ≤0.8 m/s, *EWGSOP2* predicts better mortality risk in older adults. LGS is a better mortality risk predictor only when LMS is defined using lower cut-off.

## Supplementary Material

aa-21-2110-File002_afac164
