# Supplementary material for: European Working Group on Sarcopenia in Older People 2010 (EWGSOP1) and 2019 (EWGSOP2) criteria or slowness: which is the best predictor of mortality risk in older adults?
Source: Age Ageing. 2022 Jul 23;51(7):afac164. doi: 10.1093/ageing/afac164 (PMC9338689; doi:10.1093/ageing/afac164)
Supplement: aa-21-2110-File002_afac164 [file aa-21-2110-file002_afac164.docx]

**European Working Group on Sarcopenia in Older People 2010 (EWGSOP1) and 2019 (EWGSOP2) criteria or slowness: which is the best predictor of mortality risk in older adults?**

**SUPPLEMENTARY MATERIAL**

1. Supplementary Methods
2. Supplemental Table 1. Final Cox proportional hazard models predicting mortality in 14-year follow-up among 6,182 older adults from ELSA Study
3. Supplementary References

**1. Supplementary Methods**

**Muscle strength assessment**

HGS was measured using a dynamometer (*Smedley*; range: 0 to 100 kg). The test was performed with the participant standing, arms alongside the trunk, and elbow at 90 degrees[1]. Three trials were performed using the dominant hand, with a one-minute rest interval between trials. The highest value was considered for the analysis[2,3]. HGS was used as a continuous variables in the accuracy analysis. To determine whether different HGS cut-offs used in the definitions of sarcopenia would alter the associations between sarcopenia and mortality, LMS was considered when HGS was <32, <30, <27, and <26 kg for men and <21, <20, and <16 kg for women[4–9].

**Appendicular skeletal muscle mass assessment**

Appendicular skeletal muscle mass (ASMM) was determined using the Lee equation[10]. Al-Gindan et al.[11] validated this equation using whole-body magnetic resonance imaging as the reference method and found an adjusted coefficient of determination of 0.85 for men and women. In a study investigating the association between multimorbidity at baseline and the onset of sarcopenia over 12 years of follow-up in a large representative sample of the English older adult population, Veronese et al. also used this equation to estimate ASMM[12]. After the estimation of ASMM, the appendicular skeletal muscle mass index (ASMMI) (kg/m^2^) was calculated. LMM was considered when the ASMMI was <9.24 kg/m^2^ for men and <6.52 kg/m^2^ for women. ASMMI values were defined based on the 20^th^ percentile of the sample distribution[13,14].

**Physical performance**

GS was used for the assessment of physical performance, considering the better time between two consecutive trials along a 2.4-meter track on a flat surface without the use of a gait-assistance device[15,16]. The total track in meters was divided by the time in seconds for the conversion into meters/second (m/s). GS was used as a continuous variable in the accuracy analysis. In the mortality analyses, GS ≤0.8 m/s was considered being LGS[4,6].

All measures used for the definition and diagnosis of sarcopenia were taken at baseline.

**Definition and diagnosis of sarcopenia**

The criteria proposed by the *EWGSOP1*[6] and *EWGSOP2*[4] were used for the definition of sarcopenia. Based on the definition of the *EWGSOP1*[6], individuals with LMM were considered pre-sarcopenic; LMM+LMS or LGS were considered sarcopenic; and LMM+LMS+LGS were considered severely sarcopenic. Based on the definition of the *EWGSOP2*[4], individuals with clinical suspicion and LMS were considered as having probable sarcopenia; those with LMS+LMM were considered sarcopenic; and those with LMS+LMM+LGS were considered severely sarcopenic. LMM, LMS, and LGS as isolated conditions were also analysed to identify which had a stronger association with an increased risk of mortality.

**Covariates**

The covariates included in the present analysis constitute a broad spectrum of factors associated with mortality[17–19]. The sociodemographic characteristics were sex, age, total household wealth classified in quintiles, marital status (presence/absence of conjugal life), and level of education. The English three-way education system was qualified to a level lower than “O-level” or equivalent (0-11 years of schooling), a level lower than “A-level” or equivalent (12-13 years), and a higher qualification (more than 13 years) [20,21].

The behavioural characteristics were smoking status, alcohol intake, and physical activity level. For smoking, the individuals were classified as non-smokers, ex-smokers or smokers. Regarding alcohol intake, the individuals were classified as non-drinkers or rare drinkers (once per week), frequent drinkers (2-6 times per week) or daily drinkers[20]. For physical activity, the individuals were classified as sedentary (vigorous or moderate, once per week, one to three times per month, hardly ever or never; any mild physical activity) or active (vigorous or moderate, more than once per week)[22] based on the Physical Activity and Sedentary Behaviour Assessment Questionnaire validated by the Health Survey for England[23].

Clinical conditions were identified based on self-reports of a medical diagnosis of systemic arterial hypertension, diabetes, cancer, lung disease, heart disease, stroke, and falls in the previous year. Depressive symptoms were determined using short version of the Center for Epidemiologic Studies Depression Scale (CES-D) and the risk of depression was considered when ≥4 points[24]. Memory was assessed using the Word-List Learning Test, which ranges from 0-20 words, with higher scores indicating a better memory performance[25]. Number of medications was evaluated based on self-reports of use with a medical recommendation.

Waist circumference (WC) was determined using a flexible, non-elastic metric tape positioned at the midpoint between the iliac crest and last rib with the participant standing in light clothing and arms alongside the body. WC was measured with the abdomen relaxed at the end of expiration[1]. Abdominal obesity was defined as WC >88 cm for women and >102 cm for men[26].

**Supplemental Table 1.** Final Cox proportional hazard models predicting mortality in 14-year follow-up among 6,182 older adults from ELSA Study

|  | **Construct 1** | **Construct 2** | **Construct 3** | **Construct 4** | **Construct 5** | **Construct 6** | **Construct 7** | **Construct 8** | **Construct 9** | **Construct 10** |
| --- | --- | --- | --- | --- | --- | --- | --- | --- | --- | --- |
|  | **HR (95% CI)** | **HR (95% CI)** | **HR (95% CI)** | **HR (95% CI)** | **HR (95% CI)** | **HR (95% CI)** | **HR (95% CI)** | **HR (95% CI)** | **HR (95% CI)** | **HR (95% CI)** |
| **Sociodemographic characteristics** | |  |  |  |  |  |  |  |  |  |
| Age, years | 1.10 (1.09-1.11) | 1.10 (1.09-1.11) | 1.10 (1.09-1.11) | 1.10 (1.09-1.11) | 1.10 (1.09-1.11) | 1.10 (1.09-1.11) | 1.10 (1.09-1.11) | 1.10 (1.10-1.11) | 1.10 (1.09-1.11) | 1.10 (1.09-1.10) |
| Sex, male | 1.53 (1.40-1.67) | 1.53 (1.40-1.67) | 1.53 (1.40-1.68) | 1.54 (1.41-1.68) | 1.54 (1.41-1.68) | 1.52 (1.39-1.66) | 1.52 (1.39-1.66) | 1.55 (1.41-1.69) | 1.56 (1.43-1.71) | 1.58 (1.44-1.72) |
| Marital status, without conjugal life | 1.01 (0.92-1.11) | 1.01 (0.92-1.11) | 1.01 (0.92-1.11) | 1.01 (0.92-1.11) | 1.01 (0.92-1.12) | 1.02 (0.93-1.12) | 1.02 (0.93-1.12) | 1.02 (0.93-1.12) | 1.02 (0.93-1.12) | 1.02 (0.93-1.12) |
| Total household wealth |  |  |  |  |  |  |  |  |  |  |
| 1^st^ quintile (highest quintile) | 1.00 | 1.00 | 1.00 | 1.00 | 1.00 | 1.00 | 1.00 | 1.00 | 1.00 | 1.00 |
| 2^nd^ quintile | 1.11 (0.96-1.28) | 1.11 (0.96-1.28) | 1.11 (0.96-1.28) | 1.11 (0.96-1.27) | 1.11 (0.96-1.28) | 1.11 (0.97-1.29) | 1.11 (0.96-1.29) | 1.11 (0.96-1.28) | 1.11 (0.96-1.28) | 1.11 (0.96-1.29) |
| 3^rd^ quintile | 1.20 (1.05-1.39) | 1.20 (1.05-1.39) | 1.20 (1.05-1.39) | 1.20 (1.04-1.38) | 1.20 (1.04-1.38) | 1.20 (1.04-1.38) | 1.20 (1.04-1.39) | 1.20 (1.04-1.39) | 1.20 (1.04-1.38) | 1.19 (1.03-1.37) |
| 4^th^ quintile | 1.20 (1.04-1.40) | 1.20 (1.04-1.40) | 1.21 (1.04-1.40) | 1.20 (1.03-1.39) | 1.20 (1.04-1.39) | 1.21 (1.04-1.40) | 1.21 (1.04-1.41) | 1.20 (1.03-1.39) | 1.20 (1.03-1.39) | 1.19 (1.02-1.38) |
| 5^th^ quintile (lowest quintile) | 1.32 (1.13-1.54) | 1.32 (1.13-1.54) | 1.32 (1.13-1.54) | 1.31 (1.13-1.53) | 1.31 (1.13-1.53) | 1.32 (1.13-1.53) | 1.32 (1.13-1.54) | 1.30 (1.12-1.52) | 1.30 (1.11-1.51) | 1.28 (1.09-1.49) |
| **Behavioural characteristics** |  |  |  |  |  |  |  |  |  |  |
| Smoking status |  |  |  |  |  |  |  |  |  |  |
| Never smoked | 1.00 | 1.00 | 1.00 | 1.00 | 1.00 | 1.00 | 1.00 | 1.00 | 1.00 | 1.00 |
| Former smoker | 1.21 (1.10-1.33) | 1.21 (1.10-1.33) | 1.21 (1.10-1.33) | 1.22 (1.11-1.34) | 1.22 (1.11-1.34) | 1.21 (1.10-1.33) | 1.21 (1.10-1.33) | 1.21 (1.10-1.33) | 1.21 (1.10-1.33) | 1.21 (1.10-1.33) |
| Current smoker | 2.02 (1.77-2.30) | 2.02 (1.77-2.30) | 2.02 (1.77-2.30) | 2.02 (1.77-2.31) | 2.02 (1.77-2.30) | 2.04 (1.79-2.32) | 2.04 (1.79-2.32) | 2.03 (1.78-2.32) | 2.01 (1.77-2.30) | 1.99 (1.74-2.27) |
| Alcohol intake |  |  |  |  |  |  |  |  |  |  |
| Non-drinker or rare drinker | 1.00 | 1.00 | 1.00 | 1.00 | 1.00 | 1.00 | 1.00 | 1.00 | 1.00 | 1.00 |
| Frequent drinker | 0.81 (0.72-0.91) | 0.81 (0.72-0.91) | 0.81 (0.72-0.91) | 0.81 (0.72-0.90) | 0.80 (0.72-0.90) | 0.80 (0.72-0.90) | 0.80 (0.72-0.90) | 0.80 (0.72-0.90) | 0.80 (0.71-0.89) | 0.79 (0.71-0.88) |
| Daily drinker | 0.87 (0.77-0.98) | 0.87 (0.77-0.98) | 0.87 (0.76-0.98) | 0.87 (0.76-0.98) | 0.86 (0.76-0.98) | 0.86 (0.76-0.97) | 0.86 (0.76-0.97) | 0.86 (0.76-0.97) | 0.86 (0.76-0.97) | 0.85 (0.75-0.96) |
| Activity level, sedentary | 1.27 (1.17-1.39) | 1.27 (1.17-1.39) | 1.27 (1.17-1.39) | 1.27 (1.17-1.39) | 1.27 (1.16-1.38) | 1.29 (1.18-1.41) | 1.29 (1.18-1.40) | 1.28 (1.17-1.39) | 1.27 (1.16-1.38) | 1.25 (1.15-1.37) |
| **Clinical characteristics** |  |  |  |  |  |  |  |  |  |  |
| Systemic arterial hypertension, yes | 1.04 (0.95-1.13) | 1.04 (0.95-1.13) | 1.04 (0.95-1.13) | 1.04 (0.95-1.13) | 1.03 (0.95-1.13) | 1.02 (0.93-1.11) | 1.02 (0.93-1.11) | 1.03 (0.94-1.12) | 1.03 (0.94-1.12) | 1.03 (0.94-1.12) |
| Diabetes, yes | 1.23 (1.08-1.40) | 1.23 (1.08-1.40) | 1.23 (1.08-1.40) | 1.23 (1.08-1.40) | 1.23 (1.08-1.40) | 1.22 (1.07-1.39) | 1.22 (1.07-1.39) | 1.21 (1.06-1.38) | 1.21 (1.06-1.38) | 1.19 (1.05-1.36) |
| Cancer, yes | 1.60 (1.40-1.83) | 1.60 (1.40-1.83) | 1.60 (1.40-1.83) | 1.60 (1.40-1.83) | 1.59 (1.39-1.82) | 1.59 (1.39-1.82) | 1.59 (1.40-1.82) | 1.59 (1.39-1.82) | 1.59 (1.39-1.81) | 1.55 (1.36-1.78) |
| Lung disease, yes | 1.12 (1.01-1.26) | 1.12 (1.01-1.26) | 1.13 (1.01-1.26) | 1.12 (1.01-1.26) | 1.12 (1.00-1.25) | 1.14 (1.02-1.27) | 1.14 (1.02-1.27) | 1.14 (1.02-1.27) | 1.13 (1.01-1.26) | 1.12 (1.01-1.26) |
| Heart disease, yes | 1.27 (1.16-1.39) | 1.27 (1.16-1.39) | 1.28 (1.17-1.40) | 1.27 (1.16-1.39) | 1.28 (1.17-1.40) | 1.27 (1.16-1.39) | 1.27 (1.16-1.39) | 1.26 (1.16-1.38) | 1.26 (1.15-1.38) | 1.27 (1.16-1.39) |
| Stroke, yes | 1.25 (1.09-1.43) | 1.25 (1.09-1.43) | 1.25 (1.09-1.43) | 1.25 (1.09-1.43) | 1.25 (1.09-1.43) | 1.25 (1.09-1.43) | 1.25 (1.09-1.43) | 1.25 (1.09-1.42) | 1.24 (1.08-1.42) | 1.26 (1.10-1.44) |
| Falls, yes | 1.04 (0.95-1.13) | 1.04 (0.95-1.13) | 1.04 (0.95-1.13) | 1.04 (0.95-1.13) | 1.04 (0.95-1.13) | 1.03 (0.95-1.12) | 1.03 (0.94-1.12) | 1.03 (0.94-1.12) | 1.02 (0.93-1.11) | 1.02 (0.94-1.11) |
| Depressive Symptoms, yes | 1.09 (0.98-1.22) | 1.09 (0.98-1.22) | 1.09 (0.98-1.22) | 1.09 (0.97-1.22) | 1.08 (0.97-1.21) | 1.09 (0.97-1.22) | 1.09 (0.98-1.22) | 1.09 (0.98-1.22) | 1.09 (0.98-1.22) | 1.08 (0.96-1.20) |
| Memory Score, points | 0.96 (0.95-0.97) | 0.96 (0.95-0.97) | 0.96 (0.95-0.97) | 0.96 (0.95-0.97) | 0.96 (0.95-0.97) | 0.96 (0.94-0.97) | 0.96 (0.94-0.97) | 0.96 (0.95-0.97) | 0.96 (0.95-0.97) | 0.96 (0.95-0.97) |
| Medications, number | 1.08 (1.01-1.16) | 1.08 (1.01-1.16) | 1.08 (1.01-1.16) | 1.07 (1.01-1.15) | 1.07 (1.00-1.15) | 1.08 (1.01-1.16) | 1.08 (1.01-1.16) | 1.08 (1.01-1.15) | 1.07 (1.01-1.15) | 1.07 (1.00-1.15) |
| Abdominal obesity, yes | 0.99 (0.89-1.11) | 0.99 (0.89-1.11) | 1.00 (0.89-1.11) | 1.00 (0.90-1.12) | 1.01 (0.90-1.13) | 0.90 (0.82-0.98) | 0.90 (0.82-0.99) | 0.92 (0.83-1.02) | 0.95 (0.85-1.05) | 1.00 (0.90-1.11) |
| No sarcopenia | 1.00 | 1.00 | 1.00 | 1.00 | 1.00 | 1.00 | 1.00 | 1.00 | 1.00 | 1.00 |
| Pre-sarcopenia | 1.11 (0.94-1.32) | 1.10 (0.93-1.31) | 1.11 (0.91-1.34) | 1.07 (0.86-1.33) | 0.95 (0.71-1.28) | 1.06 (0.90-1.24) | 1.03 (0.90-1.18) | 1.08 (0.95-1.23) | 1.12 (1.00-1.26) | 1.30 (1.16-1.46) |
| Sarcopenia | 1.37 (1.17-1.61) | 1.38 (1.17-1.63) | 1.32 (1.12-1.56) | 1.26 (1.06-1.49) | 1.23 (1.05-1.45) | 1.02 (0.73-1.42) | 1.06 (0.77-1.45) | 1.08 (0.86-1.36) | 1.21 (0.99-1.48) | 1.48 (1.24-1.78) |
| Severe Sarcopenia | 1.36 (1.11-1.67) | 1.36 (1.12-1.65) | 1.37 (1.16-1.61) | 1.43 (1.22-1.68) | 1.48 (1.27-1.73) | 1.21 (0.97-1.50) | 1.19 (0.95-1.50) | 1.29 (1.08-1.53) | 1.44 (1.21-1.71) | 1.78 (1.49-2.12) |

HR: Hazard Ratio. CI: Confidence Interval. Education level was not included in final model due high collinearity with wealth.

***EWGSOP1*:**

Construct 1- low muscle strength < 26/16 kg

Construct 2- low muscle strength < 27/16 kg

Construct 3- low muscle strength < 30/20 kg

Construct 4- low muscle strength < 32/21 kg

Construct 5- low muscle strength < 36/23 kg

***EWGSOP2*:**

Construct 6- low muscle strength < 26/16 kg

Construct 7- low muscle strength < 27/16 kg

Construct 8- low muscle strength < 30/20 kg

Construct 9- low muscle strength < 32/21 kg

Construct 10- low muscle strength < 36/23 kg

**3. Supplementary References**

1. Banks J, Breeze E, Lessof C NJ. Retirement, health and relationships of the older population in England: the 2004 English Longitudinal Study of Ageing. London: Institute for Fiscal Studies; 2006.

2. Al Snih S, Markides KS, Ottenbacher KJ, Raji MA. Hand grip strength and incident ADL disability in elderly Mexican Americans over a seven-year period. Aging clinical and experimental research. 2004 Dec;16(6):481–6.

3. Bohannon RW, Magasi S. Identification of dynapenia in older adults through the use of grip strength t-scores. Muscle & nerve. 2015 Jan;51(1):102–5.

4. Cruz-Jentoft AJ, Bahat G, Bauer J, Boirie Y, Bruyère O, Cederholm T, et al. Sarcopenia: Revised European consensus on definition and diagnosis. Age and Ageing. 2019.

5. Dodds RM, Syddall HE, Cooper R, Benzeval M, Deary IJ, Dennison EM, et al. Grip strength across the life course: Normative data from twelve British studies. PLoS ONE. 2014;

6. Cruz-Jentoft AJ, Baeyens JP, Bauer JM, Boirie Y, Cederholm T, Landi F, et al. Sarcopenia: European consensus on definition and diagnosis. Age and Ageing. 2010;39(4):412–23.

7. Lauretani F, Russo CR, Bandinelli S, Bartali B, Cavazzini C, Di Iorio A, et al. Age-associated changes in skeletal muscles and their effect on mobility: an operational diagnosis of sarcopenia. Journal of applied physiology (Bethesda, Md : 1985). 2003 Nov;95(5):1851–60.

8. Delinocente MLB, de Carvalho DHT, Máximo R de O, Chagas MHN, Santos JLF, Duarte YA de O, et al. Accuracy of different handgrip values to identify mobility limitation in older adults. Archives of gerontology and geriatrics. 2021;94:104347.

9. Alley DE, Shardell MD, Peters KW, McLean RR, Dam T-TL, Kenny AM, et al. Grip strength cutpoints for the identification of clinically relevant weakness. The journals of gerontology Series A, Biological sciences and medical sciences. 2014 May;69(5):559–66.

10. Lee, Robert C, Wang, ZiMian, Heo, Moonseong, Ross, Robert, Janssen, Ian, Heymsfield SB. Total-body skeletal muscle mass: Development and cross-validation of anthropometric prediction models. American Journal of Clinical Nutrition. 2000;72(3):796–803.

11. Al-Gindan YY, Hankey C, Govan L, Gallagher D, Heymsfield SB, Lean MEJ. Derivation and validation of simple equations to predict total muscle mass from simple anthropometric and demographic data. The American journal of clinical nutrition. 2014 Oct;100(4):1041–51.

12. Veronese N, Smith L, Cereda E, Maggi S, Barbagallo M, Dominguez LJ, et al. Multimorbidity increases the risk for sarcopenia onset: Longitudinal analyses from the English Longitudinal Study of Ageing. Experimental gerontology. 2021 Dec;156:111624.

13. Delmonico MJ, Harris TB, Lee JS, Visser M, Nevitt M, Kritchevsky SB, et al. Alternative definitions of sarcopenia, lower extremity performance, and functional impairment with aging in older men and women. Journal of the American Geriatrics Society. 2007;55(5):769–74.

14. Coin A, Sarti S, Ruggiero E, Giannini S, Pedrazzoni M, Minisola S, et al. Prevalence of Sarcopenia Based on Different Diagnostic Criteria Using DEXA and Appendicular Skeletal Muscle Mass Reference Values in an Italian Population Aged 20 to 80. Journal of the American Medical Directors Association [Internet]. 2013;14(7):507–12. Available from: http://dx.doi.org/10.1016/j.jamda.2013.02.010

15. Guralnik JM, Simonsick EM, Ferrucci L, Glynn RJ, Berkman LF, Blazer DG, et al. A short physical performance battery assessing lower extremity function: association with self-reported disability and prediction of mortality and nursing home admission. Journal of gerontology. 1994 Mar;49(2):M85-94.

16. Guralnik JM, Ferrucci L, Pieper CF, Leveille SG, Markides KS, Ostir G V, et al. Lower extremity function and subsequent disability: consistency across studies, predictive models, and value of gait speed alone compared with the short physical performance battery. The journals of gerontology Series A, Biological sciences and medical sciences. 2000 Apr;55(4):M221-31.

17. Petermann-Rocha F, Chen M, Gray SR, Ho FK, Pell JP, Celis-Morales C. New versus old guidelines for sarcopenia classification: What is the impact on prevalence and health outcomes? Age and ageing. 2020 Feb;49(2):300–4.

18. Bachettini NP, Bielemann RM, Barbosa-Silva TG, Menezes AMB, Tomasi E, Gonzalez MC. Sarcopenia as a mortality predictor in community-dwelling older adults: a comparison of the diagnostic criteria of the European Working Group on Sarcopenia in Older People. European journal of clinical nutrition. 2020 Apr;74(4):573–80.

19. Xu J, Wan CS, Ktoris K, Reijnierse EM, Maier AB. Sarcopenia Is Associated with Mortality in Adults: A Systematic Review and Meta-Analysis. Gerontology. Switzerland; 2021. p. 1–16.

20. Alexandre T da S, Scholes S, Ferreira Santos JL, Duarte YA de O, de Oliveira C. The combination of dynapenia and abdominal obesity as a risk factor for worse trajectories of IADL disability among older adults. Clinical nutrition (Edinburgh, Scotland). 2018 Dec;37(6 Pt A):2045–53.

21. Banks J, Kumari M, Smith JP, Zaninotto P. What explains the American disadvantage in health compared with the English? The case of diabetes. Journal of epidemiology and community health. 2012 Mar;66(3):259–64.

22. Alexandre T da S, Scholes S, Santos JLF, de Oliveira C. Dynapenic Abdominal Obesity as a Risk Factor for Worse Trajectories of ADL Disability Among Older Adults: The ELSA Cohort Study. The journals of gerontology Series A, Biological sciences and medical sciences. 2019 Jun;74(7):1112–8.

23. Rivilis I, Hay J, Cairney J, Klentrou P, Liu J FB. Joint health surveys unit, National Centre for social research and University College London research Department of Epidemiology and Public Health. The health survey for England 2008. Res Dev Disabil. 2011;32:894–910.

24. Radloff LS. The CES-D scale. Appl Psychol Meas. 1977;1:385–401.

25. Steel N, Huppert FA, McWilliams B MD. Physical and cognitive function. In: Marmot M, Banks J, Blundell R, Lessof C, Nazroo J, editors. Health, Wealth and Lifestyles of the Older Population in England: The 2002 English Longitudinal Study of Ageing. 2003. p. 249–300.

26. Clinical Guidelines on the Identification, Evaluation, and Treatment of Overweight and Obesity in Adults--The Evidence Report. National Institutes of Health. Obesity research. 1998 Sep;6 Suppl 2:51S-209S.
